# A three-armed cognitive-motor exercise intervention to increase spatial orientation and life-space mobility in nursing home residents: study protocol of a randomized controlled trial in the PROfit project

**DOI:** 10.1186/s12877-020-01840-0

**Published:** 2020-10-31

**Authors:** Bettina Wollesen, Madeleine Fricke, Carl-Philipp Jansen, Katharina Gordt, Michael Schwenk, Thomas Muehlbauer, Christina Morawietz, Adele Kruse, Klaus Gramann

**Affiliations:** 1 Department of Biological Psychology and Neuroergonomics, TU Berlin, Fasanenstr. 1, 10623 Berlin, Germany; 2 Department of Human Movement Science, University of Hamburg, Mollerstraße 10, 20148 Hamburg, Germany; 3 Network Aging Research, Heidelberg University, Bergheimer Str. 20, 69115 Heidelberg, Germany; 4 Institute of Sports and Sports Sciences, Heidelberg University, Im Neuenheimer Feld 720, 69120 Heidelberg, Germany; 5 Division of Movement and Training Sciences/Biomechanics of Sport, University of Duisburg-Essen, Gladbecker Str. 182, 45141 Essen, Germany; 6 School of Software, University of Technology Sydney, Sydney, 2007 Australia

**Keywords:** Nursing home, Multi-modal intervention, Spatial navigation, Cognitive functioning, Physical activity, Life-space

## Abstract

**Background:**

In nursing home residents, the combination of decreasing mobility and declining cognitive abilities, including spatial orientation, often leads to reduced physical activity (PA) and life-space (LS) mobility. As a consequence of sedentary behavior, there is a lack of social interaction and cognitive stimulation, resulting in low quality of life. It has not yet been examined whether cognitive-motor training including spatial cognitive tasks is suitable to improve spatial orientation and, as a consequence, to enlarge LS mobility, and increase well-being and general cognitive-motor functioning. Therefore, the overall goal of this multicentric randomized controlled trial (RCT) is to compare the effect of three different intervention approaches including functional exercise and orientation tasks on PA, LS and spatial orientation in nursing home residents.

**Methods:**

A three-arm single-blinded multicenter RCT with a wait-list control group will be conducted in a sample of 513 individuals (needed according to power analysis) in three different regions in Germany. In each nursing home, one of three different intervention approaches will be delivered to participating residents for 12 weeks, twice a week for 45 min each: The *PROfit basic* group will perform functional strength, balance, flexibility, and walking exercises always at the same location, whereas the *PROfit plus* group changes the location three times while performing similar/the same exercises as the PROfit basic group. The *PROfit orientation* group receives navigation tasks in addition to the relocation during the intervention. Physical and cognitive functioning as well as psychological measures will be assessed in all study groups at baseline. Participants will then be randomized into either the intervention group or the wait-list control group. After 12 weeks, and after 24 weeks the measures will be repeated.

**Discussion:**

This study evaluates whether the three different interventions are feasible to reduce the decline of or even improve PA, LS, and spatial orientation in nursing home residents. By adding different training locations in *PROfit plus*, the program is expected to be superior to *PROfit basic* in increasing physical and cognitive parameters*.* Moreover, we expect the *PROfit orientation* intervention to be most effective in terms of PA, LS, and spatial orientation due to two mechanisms: (1) increased physical and cognitive activity will enhance cognitive-motor capacity and (2) the spatial training will help to build up cognitive strategies to compensate for age-related loss of spatial orientation abilities and related limitations.

**Trial registration:**

The trial was prospectively registered at DRKS.de with registration number *DRKS00021423** on April 16, 2020* and was granted permission by the Technical University Berlin local ethics committee (No. GR_14_20191217).

## Background

Nursing home residents frequently suffer from multimorbidity [1]. Especially in this high-aged population, progressive decline in motor and cognitive abilities often results in decreased quality of life [2, 3]. A large proportion of nursing home residents are physically inactive [4, 5] and spend their time alone [6]. Such physical inactivity has multiple negative consequences on health and well-being [7, 8]. Cognitive decline is a risk factor for reduced activities of daily living (ADL) and instrumental activities of daily living (IADL) in older adults [9, 10], which further contribute to a decreased quality of life. For example, declining cognitive abilities lead to functional limitations in performing fundamental physical and cognitive activities such as climbing stairs and producing intelligible speech [11].

It has been shown that the combination of decreasing mobility and declining cognitive abilities leads to an accelerated reduction of physical activity (PA) and life space (LS) [12–15]. Sedentary behavior hampers social interaction which in turn reduces cognitive stimulation [8]. Given the interplay between PA and cognitive abilities [16, 17], sedentary behavior aggravates cognitive impairment, leading to a vicious cycle. Beside these general impairments of cognitive abilities, age-related cognitive decline specifically affects spatial-cognitive abilities such as spatial orientation and navigation [18]. Decrements in these specific cognitive subdomains are early biomarkers of cognitive decline [19–21], with an adverse impact on mobility and, as a consequence, reduced LS mobility and PA.

LS mobility is a measure that has been positively associated with PA as well as social participation in nursing home residents [22] and – per definition – it also incorporates the use of someone’s spatial environment [23]. It was also shown to be positively associated with better cognitive function [24] and a predictor of cognitive deterioration [25, 26]. Spatial abilities underlying orientation in and navigation through the environment include remembrance of target localisation in an environment, awareness of distance and directions as well as the mental transformation of the relation between objects to own body positions and spatial orientation [27]. Spatial abilities are determined by factors of the individual’s lifespan development in various environments [28, 29] and decreasing spatial cognitive abilities can lead to reduced PA and a decreased LS mobility due to spatial anxiety [30]. However, not all spatial abilities in the aging population are affected to the same degree. To distinguish between general spatial abilities and spatial abilities that decline with increasing age, it is necessary to differentiate between egocentric and allocentric reference frames [31]. Within an egocentric reference frame, the environment is represented with regards to the current position and orientation of the person (e.g. the third door on the right leads to the dining room). Allocentric reference frames, in contrast, are centered outside the person (e.g. the dining room is located in the middle of the east wing of the building) and the spatial information is thus coded independently of the position and orientation of the person. While egocentric orientation strategies remain relatively stable in older age, allocentric strategies decrease considerably with increasing age [3, 32–34]. It seems that this reduction does not depend on individual preferences in using spatial orientation strategies across the life span [35]. During the last years, however, it has been repeatedly shown that the aging brain and body remain plastic and that older adults’ capacity can be improved through systematic motor or cognitive training [36–38]. By adding physical training components, cognitive resources can be addressed more effectively and/or flexibly [39]. Moreover, different types of dual- or multi-task training, for example combining motor exercises with unspecific orientation tasks, might positively influence the cognitive performance of older adults [37, 40]. These studies are complemented by psychological theories, for example, the resource theory [41], predicting that well-trained older adults have more resources available to perform cognitive tasks on a higher level. There is also evidence that specific training interventions will initiate mental stimulation and that mental compensation (e.g. through specific cognitive training like the method of loci) can enhance neural plasticity [38, 42].

However, some studies show that dual-task training is not always more beneficial than e.g. multicomponent exercises to gain positive effects on cognitive-motor performance in older adults [43–45]. To become more effective in improving dual-task performance, intervention programs should include a combination of complex balance and coordination tasks [46]. To optimize benefits for both, cognitive and motor functions, training interventions need to be task-specific [40, 47]. It has been shown that exercise programs including cognitive-motor elements (for example, using dual-tasking with specific muscle strengthening elements [48] to improve ADL components [49]) are more successful than cognitive or motor training provided separately. In addition, a recent study by Bherer and colleagues [50] emphasized the synergistic effects of the combination of cognitive and motor training. To achieve cognitive improvements through motor training, adaptation to participants’ individual prerequisites is important [39]. Adaptable exercise modalities are required to align exercises to individual requirements, which would thus allow for a comparable training intensity for all participants [39]. Hence, frequency, intensity, and duration have to be controlled. A recent meta-analysis revealed that cognitive-motor training should be provided for at least twelve weeks with a minimum of 60 min per week to improve executive functions [46]. Moreover, a progression that allows individual adaptions should be provided [39, 46].

The aforementioned vicious cycle of decreasing mobility and general cognitive decline can be addressed by cognitive-motor training. Previous studies that investigated training of motor function [51, 52] or spatial orientation [53, 54] demonstrated improvements in the physical and cognitive domain. Such interventions could enhance PA and LS mobility [55, 56], which may also stimulate overall cognitive performance. Moreover, Cassilhas and colleagues found that physical exercise (aerobic and resistance) improved spatial learning and memory [57]. However, it remains unclear whether these programs might induce stronger effects on cognitive-motor performance if specific cognitive components had been integrated [40]. In addition, animal studies on neurogenesis provided evidence that PA in combination with activity in cognitively enriched environments induces additive neurogenic effects in the hippocampus, an important underlying neural structure of human memory [58] and allocentric spatial orientation [59, 60]. Garthe and colleagues (2016) concluded that these findings underpin the physiological link between locomotion and orientation. Therefore, interacting with an enriched environment benefits cognitive functioning, including learning and memory abilities [61]. Moving through an enriched environment might specifically foster spatial learning as a means to enable individuals to re-orientate when confronted with the same environment [62, 63]. Moreover, in the context of long-term care Vance and colleagues [64] addressed different methods of cognitive training interventions (e.g. method of loci) to improve cognitive abilities such as orientation abilities or compensation strategies for the loss or limitation of cognitive strategies [64]. However, these methods have not yet become a standardized part of cognitive-motor interventions. One of the specific exercise components which have not been addressed in particular in nursing home residents is spatial orientation. It can be addressed via two different strategies within a training program. Firstly, physical training intervention can include dual-task elements addressing egocentric and allocentric aspects of spatial orientation. Secondly, integrating supportive spatial information like landmarks into care facilities is another means to compensate for decreasing spatial cognitive functions and to support the spatial orientation of nursing home residents [65]. In addition to landmarks that are given because of their functionality within the facility (e.g. social rooms, restaurant, etc.) or that are part of the immediate environment of the facility (trees, fountains, next bus stop, etc.), these supportive landmarks can be used within a training intervention to foster spatial learning based on distinct spatial reference frames.

It has not yet been examined whether cognitive-motor training including spatial orientation tasks is suitable to increase PA, enlarge LS mobility and to improve spatial orientation and, as a consequence, elicit changes in well-being and general cognitive-motor functioning in nursing home residents. Against this background, the overall goal of this multicentric RCT (PROfit) is to investigate the efficacy of three different intervention approaches on PA, LS mobility, and spatial orientation: The *PROfit basic* group will perform functional strength, balance, flexibility, and walking exercises at the same location, while the *PROfit plus* group relocates three times during the training. The *PROfit orientation* group receives navigation tasks in addition to the relocation during interventions. We hypothesize that all three intervention arms will generate slower decline or even improvements in residents’ PA, LS mobility, and spatial orientation compared to a wait-list control group. We assume that *PROfit plus* will be more effective than *PROfit basic* because of residents’ improved orientation due to the different training locations. Furthermore, we expect the *PROfit orientation* intervention to be most beneficial due to its additional focus on spatial orientation.

## Methods/study design

This protocol paper was drafted according to the SPIRIT statement [66].

### Trial design

Based on the existing research and the physical training recommendations mentioned above, a multicenter intervention study will be conducted, aiming to determine the feasibility and efficacy of three different exercise intervention programs for residents of nursing homes. The type of intervention program will be randomly assigned to the participating nursing homes. The participants’ allocation to the intervention or wait-list control group will be randomized after baseline assessment in each nursing home. The assessment of primary and secondary outcomes will take place upon entry to the study (T1) by a blinded assessor and will be repeated at twelve (T2) and at 24 weeks (T3) (see Table 1).

**Table 1:**
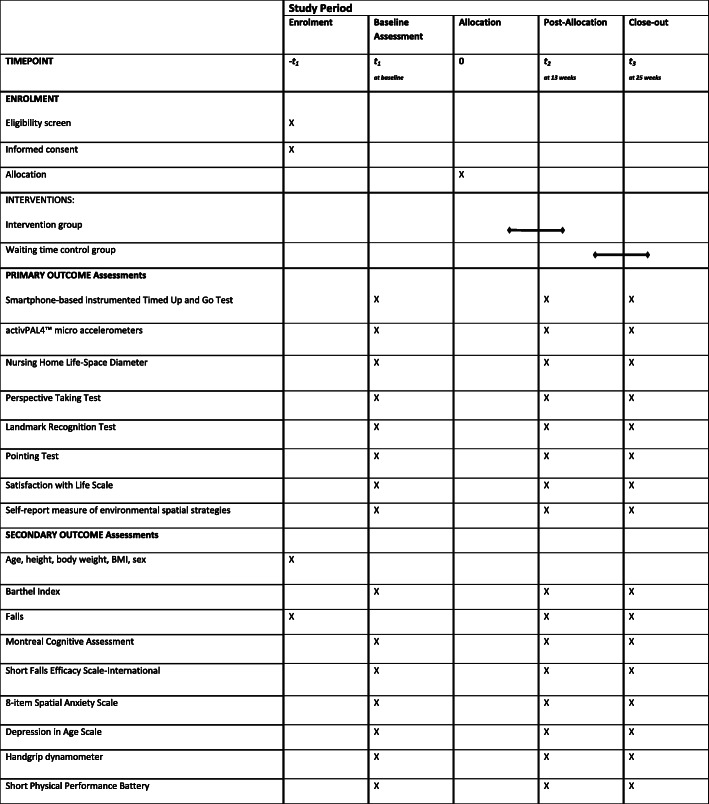
Schedule of enrolment, interventions, and assessments

### Participants, interventions, and outcomes

#### Ethical approval

The multicentric RCT is conducted in agreement with the principles of the Declaration of Helsinki and the guidelines of Good Clinical Practice. Written informed consent will be obtained from all participants or their legal guardians before enrolment in the study. The local ethics committee of the TU Berlin, Germany has approved the study protocol *(No GR_14_20191217).* The trial was registered at DRKS.de with registration number DRKS00021423 on April 16, 2020.

#### Recruitment of participants

To assure eligibility and recruitment of participants, staff consultation and nursing documentation will be applied primarily. To gain a sufficient number of participants, the institutions involved are deliberately selected based on their number of nursing places (> 100).

##### Eligibility criteria

Inclusion criteria are *i)* willingness to participate, *ii)* ability to participate in group activities, *iii)* ability to walk (with or without walking aid), and *iv)* the ability to understand and execute simple instructions including visual presentations of landmarks. No other inclusion or exclusion criteria will be applied.

#### Assignment of interventions

To avoid selection bias, stratified randomization will be conducted to divide participants into an intervention group and a wait-list control group. The random allocation will be stratified according to sex, age, and cognitive performance to avoid differences in the baseline conditions between the groups. Data collection will be done by blinded assessors; data will be stored securely and in pseudonymized form using a coded ID number to maintain participants’ confidentiality. To avoid performance bias, the measurements and the interventions follow a standardized protocol.

### Outcome measures

The assessment will focus on three key domains: physical functioning (especially LS mobility), cognitive performance (especially spatial orientation), and psychosocial well-being. Apart from the following primary and secondary outcomes, demographic and baseline characteristics, such as chronological age, body height, body mass, body mass index, and sex will be assessed.

#### Primary outcomes

**Fig. 1:**
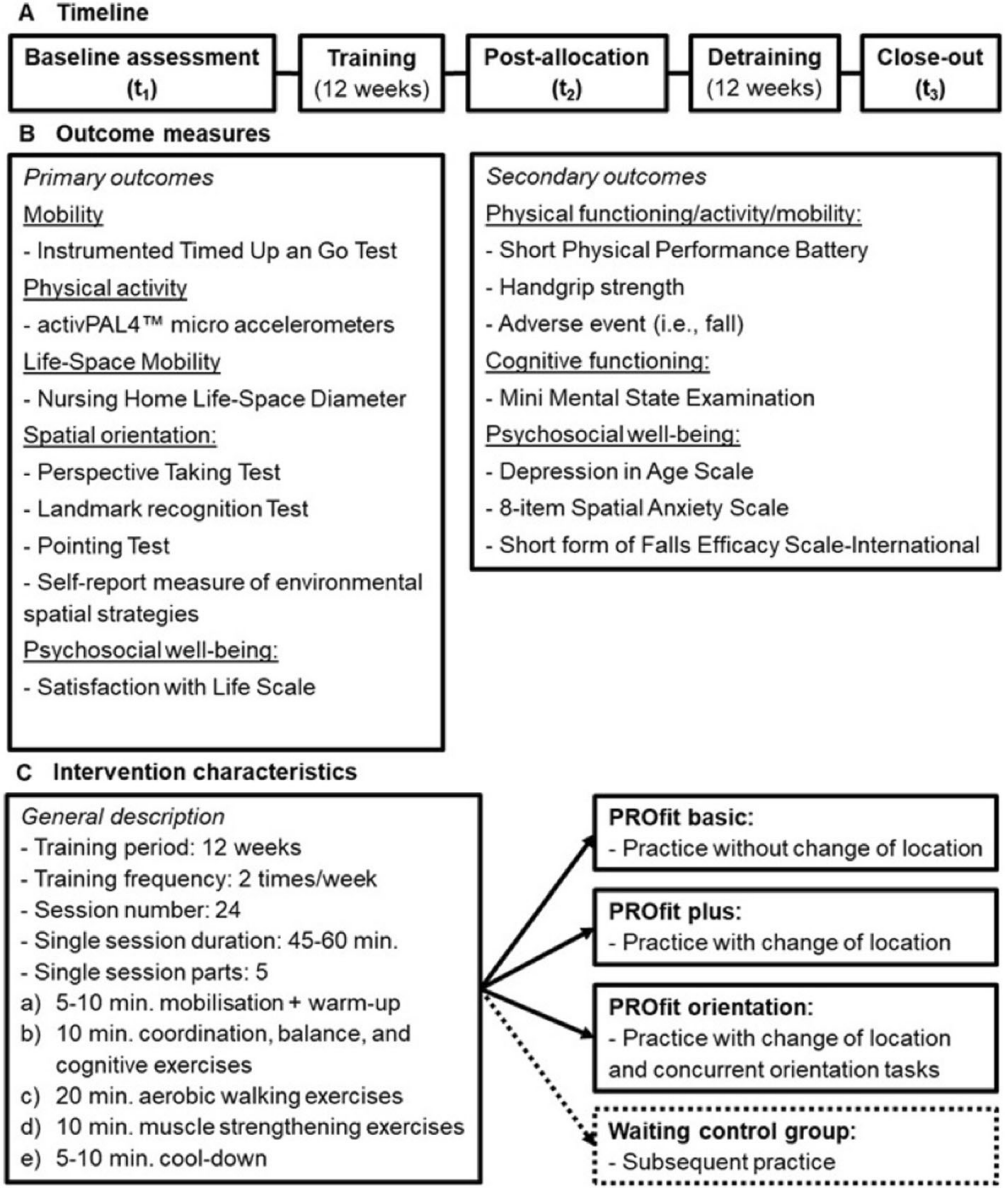
Schematic description of the study design, the outcome measures, and the intervention characteristics

The following primary outcomes will be measured to evaluate the efficacy of the intervention programs (see Fig. 1):

##### Mobility

The smartphone-based instrument Timed Up and Go Test (iTUG) is a reliable measure of mobility [67]. The instrumented TUG takes 5–10 min. The test includes two repetitions of the same task, the participants are asked to rise in a comfortable and safe pace from a chair, walk three meters, turn around, walk back to the chair and sit down.

##### Physical activity

The “activPAL4™ micro” accelerometers (PAL Technologies Ltd., Glasgow, Scotland) will be worn to assess participants’ PA for a full 7 days. The main outcomes are the number of steps as well as the proportion of PA and sedentariness per day. The reliability was judged as good to excellent (inter-device reliability range = 0.79 to 0.99) and it is a valid measure for posture and motion during everyday PA [68].

##### Life-space mobility

The Nursing Home Life-Space Diameter (NHLSD) represents an external measure of LS mobility among nursing home residents [69]. This proxy-assessment will be administered to nursing staff and can be used to monitor changes or the effect of interventions on LS mobility [69]. The interrater (Pearson’s r = 0.951) and the intrarater (Pearson’s r = 0.922) reliability of the NHLSD is excellent [69]. The extent of LS mobility is measured by dividing the nursing home into four zones: (1) the resident’s private room, (2) the ward on which she/he resides, (3) the rest of the facility beyond the ward, (4) and the area outside of the facility [69]. Frequency of travels to each of the four zones is grouped into 0 = never, 1 = less than weekly, 2 = at least weekly, 3 = > 2 times a week, 4 = 1–3 times a day, and 5 = > 3 times a day; this score is then multiplied with 1 to 4 (congruent with the value of the zones, 1 to 4) and summed up. Independence of movement can be incorporated into the overall score by multiplying it with two in case of travel without human assistance.

##### Spatial orientation

The Perspective Taking Test is a reliable (Cronbach’s alpha = .82) and valid measure of spatial orientation [70]. It contains a sheet of paper with a consideration of seven objects, which is visible at all times. Each participant gets twelve task sheets with a circle on them. For each task the participants are asked to imagine being at the position of the object in the center of the circle, facing another object, and are asked to specify the direction to a third object [71]. The number of examples, the instructions, and the operation of the test is adapted to the target group of nursing home residents.

To assess landmark recognition, we will adapt the tasks conducted by Deipolyi and colleagues [72], which were already used with cognitively impaired older adults. In this test, participants have to identify which three landmarks out of a set of ten landmark pictures cannot be found in or around the facility. The pictures show landmarks that can be located in the participant’s own room, on the floor level of their own room, inside the facility or outside, close to the facility. Afterwards, the participants have to sort the pictures showing landmarks from their facility by distance, starting from their current position. As the last step, they position the landmarks on an overview map of the facility and surroundings.

Furthermore, a pointing task will be used to ask participants to point to their own room and to six other landmarks in or around the facility, which are also used for the landmark recognition task. Bryant [73] reports that pointing errors significantly correlate with self-ratings of sense of direction (r = − 0.63), worrying about becoming lost (r = 0.51), and mental rotation (r = − 0.39).

Besides, we will assess subjective navigation abilities by a modified 19-item self-report measure of environmental spatial strategies [74]. The questionnaire reflects three different aspects of spatial orientation in a five-point Likert scale, “1” representing “totally agree” and “5” representing “totally disagree”. Self-reported navigational abilities are associated with the use of strategies for finding the way [75]. Several studies identified significant relations between self-reported estimations of spatial orientation and the actual performance in environmental tasks [73, 76–79]. For the application in nursing home residents, the items were shortened and simplified, and the situations described were adapted to the lifestyles of the respondents.

##### Psychosocial well-being

The Satisfaction with Life Scale (SWLS) [80] is a brief instrument with five items to measure global cognitive judgements of satisfaction with one’s life on a seven-point Likert scale. A meta-analysis indicated moderate results for the internal reliability of SWLS (Cronbach’s alpha = .78) [81].

#### Secondary outcomes

The following secondary outcomes will be evaluated to further assess the efficacy of the intervention programs (see Fig. 1):

##### Physical functioning

The Short Physical Performance Battery (SPPB) [82] is a standardized instrument to measure the functionality of the lower extremities (balance, gait speed, leg strength). The test battery contains three tasks: first, the participants are asked to stand upright in three different standing positions (Romberg stance, semi-tandem stance, tandem stance) for maximum 10 s each. Afterwards they complete a 4 meter walk in comfortable gait speed, the time required is measured. After that, participants are instructed to stand up and sit down for five times and as fast as possible. The score ranges for each of these tasks between zero and four points; SPPB overall scores range from zero (low mobility) to twelve (full mobility). Improvements have been demonstrated to be clinically relevant from 0.99 points for the SPPB [82]. A hydraulic hand dynamometer (JAMAR) measures hand grip strength. Two trials with each hand will be executed. The highest value of the two trials will be used for analysis.

##### Cognitive functioning

The Montreal Cognitive Assessment (MoCA) [83] is a one-page 30-items test developed for screening of mild cognitive impairment. It includes items to assess a range of cognitive domains including executive functions, visuospatial abilities, language, attention, working memory, abstraction, and orientation to time and place. The internal reliability of the MoCA is good (Cronbach’s alpha = 0.84) [84].

##### Psychosocial well-being

The Depression in Age Scale was validated for people with and without cognitive impairment living in residential care [85]. With internal reliability above 0.821 (Cronbach’s alpha), it is a reliable measure [86]. It consists of ten items.

The eight-item Spatial Anxiety Scale reliably assesses activity-related anxiety (Cronbach’s alpha = .80) [27, 87]. The short form of the Falls Efficacy Scale-International (Short-FES-I) is a reliable (Cronbach’s alpha = .89) [88] seven-item questionnaire with a scoring range between one and four. The scores of all items are summed resulting in a total score range from seven to 28, with a higher score indicating greater concern about the possibility of falling [89].

### Interventions

To develop and verify the effect of tailored interventions as well as to meet the criteria of German health insurances [90], several steps are necessary:

- Analyzing daily mobility behavior of the nursing home residents
- Examining the facilities and relevant landmarks
- Capturing the wishes and needs for training interventions and activities of the participants as well as barriers for mobility in the specific settings
- Identifying landmarks and structural elements for daily living in the facilities
- Integrating a)-d) into the interventions
- Conducting a training curriculum for future trainers in elderly care to gain sustainability
- Verifying the effects on cognitive abilities (especially spatial orientation), physical functioning and psychosocial well-being.

The exercise programs consist of 24 sessions of 45–60 min, conducted twice per week over a period of twelve weeks in groups of up to 15 participants. Exercise sessions will be administered by at least one certified exercise scientist or physiotherapist. The program follows the International Association of Gerontology and Geriatrics (IAGG) guidelines and combines previously published exercises that have proven to be beneficial for cognitive-motor performance in older people in the community and need of care [91, 92].

Based on the previous PROCARE intervention [93], three different programs will be conducted (see Table 2):

**Table 2:** Description of the intervention (modified according to Cordes and colleagues [93])

Program	Session 1–4	Session 5–8	Session 9–12	Session 13–16	Session 17–20	Session 21–24
***PROfit basic***						
Mobilisation and warm-up (5–10 min.)	e.g., range of motion exercises for the wrists, hips, shoulders, knees, and ankles	Cf. session 1–4				
Coordination, balance, and cognitive exercises (10 min.)	e.g., standing balance, bodyweight shifting, motivational cognitive motor games with group interaction including balls and scarfs	Cf. session 1–4	e.g., standing balance with feet together, side-by-side, bodyweight shifting, motivational cognitive-motor games with group interaction including balls and scarfs	Cf. session 9–12	e.g., standing balance with feet together, side-by-side, semi-tandem, tandem, standing on one leg, bodyweight shifting, motivational cognitive-motor games with group interaction including balls and scarfs	Cf. session 17–20
Aerobic walking exercises (20 min.)	e.g., under different single and dual-task conditions, 150 m	Cf. session 1–4, 150–180 m	Cf. session 1–4, 180–210 m	Cf. session 1–4, 210–240 m	Cf. session 1–4, 240–270 m	Cf. session 1–4, 270–300 m
Muscle strengthening exercises (10 min.)	e.g., chair rises, upper body and trunk exercises with additional materials and weights, functional lower-limb exercises	Cf. session 1–4	Cf. session 1–4, extended by individual adjustments of the repetition numbers	Cf. session 9–12	Cf. session 9–12, extended by individual adjustments of the weights	Cf. session 17–20
Cool down (5–10 min.)	e.g., stretching and relaxing exercises	Cf. session 1–4				
***PROfit plus***						
Mobilisation and warm-up (5–10 min.)	e.g., range of motion exercises for the wrists, hips, shoulders, knees, and ankles	Cf. session 1–4				
**Location change 1 (6–8 min.)**	e.g., under different single and dual-task conditions, 50 m	Cf. session 1–4, 50–60 m	Cf. session 1–4, 60–70 m	Cf. session 1–4, 70–80 m	Cf. session 1–4, 80–90 m	Cf. session 1–4, 90–100 m
Coordination, balance, and cognitive exercises (10 min.)	e.g., standing balance, bodyweight shifting, motivational cognitive motor games with group interaction including balls and scarfs	Cf. session 1–4	e.g., standing balance with feet together, side-by-side, bodyweight shifting, motivational cognitive-motor games with group interaction including balls and scarfs	Cf. session 9–12	e.g., standing balance with feet together, side-by-side, semi-tandem, tandem, standing on one leg, bodyweight shifting, motivational cognitive-motor games with group interaction including balls and scarfs	Cf. session 17–20
**Location change 2 (6–8 min.)**	e.g., under different single and dual-task conditions, 50 m	Cf. session 1–4, 50–60 m	Cf. session 1–4, 60–70 m	Cf. session 1–4, 70–80 m	Cf. session 1–4, 80–90 m	Cf. session 1–4, 90–100 m
Muscle strengthening exercises (10 min.)	e.g., chair rises, upper body and trunk exercises with additional materials and weights, functional lower-limb exercises	Cf. session 1–4	Cf. session 1–4, extended by individual adjustments of the repetition numbers	Cf. session 9–12	Cf. session 9–12, extended by individual adjustments of the weights	Cf. session 17–20
**Location change 3 (6–8 min.)**	e.g., under different single and dual-task conditions, 50 m	Cf. session 1–4, 50–60 m	Cf. session 1–4, 60–70 m	Cf. session 1–4, 70–80 m	Cf. session 1–4, 80–90 m	Cf. session 1–4, 90–100 m
***PROfit orientation***						
Mobilisation and warm-up, (5–10 min.)	e.g., range of motion exercises for the wrists, hip, shoulders, knees, and ankles, integrated awareness developing for cardinal points and landmarks with visual aids	Cf. session 1–4	Cf. session 1–4, reduction of the number of optical aids	Cf. session 9–12	Cf. session 9–12, removal of optical aids	Cf. session 17–20
**Location change 1 (6–8 min.)**	Direction pointing task (individually), Landmark recognition task and Landmark sequence task (group-wise), 50 m	Cf. session 1–4, 50–60 m	Cf. session 1–4, 60–70 m	Cf. session 1–4, 70–80 m	Cf. session 1–4, 80–90 m	Cf. session 1–4, 90–100 m
Coordination, balance, and cognitive exercises, (10 min.)	e.g., standing balance, bodyweight shifting, motivational cognitive motor games with group interaction including balls and scarfs, integrated pointing to cardinal directions, assembling sections of facility map, listing the sequence of landmarks within the unit	Cf. session 1–4	Cf. session 1–4, extended number of facility-sections to assemble, landmarks to arrange in sequence from the entire facility	Cf. session 9–12	Cf. session 9–12, extended number of facility-sections to assemble, landmarks to arrange in sequence from outside the facility	Cf. session 17–20
**Location change 2 (6–8 min.)**	Direction pointing task (individually), Landmark recognition task and Landmark sequence task (group-wise), 50 m	Cf. session 1–4, 50–60 m	Cf. session 1–4, 60–70 m	Cf. session 1–4, 70–80 m	Cf. session 1–4, 80–90 m	Cf. session 1–4, 90–100 m
Muscle strengthening exercises (10 min.)	e.g., chair rises, upper body and trunk exercises with additional materials and weights, functional lower-limb exercises	Cf. session 1–4	Cf. session 1–4, extended by individual adjustments of the repetition numbers	Cf. session 9–12	Cf. session 9–12, extended by individual adjustments of the weights	Cf. session 17–20
**Location change 3 (6–8 min.)**	Direction pointing task (individually), Landmark recognition task and Landmark sequence task (group-wise), 50 m	Cf. session 1–4, 50–60 m	Cf. session 1–4, 60–70 m	Cf. session 1–4, 70–80 m	Cf. session 1–4, 80–90 m	Cf. session 1–4, 90–100 m
Cool down (5–10 min.)	e.g., stretching and relaxing exercises	Cf. session 1–4

1. *PROfit basic*

*PROfit basic* focusses on daily situations which are commonly associated with increased fall risk. It mainly consists of challenging walking exercises (e.g. brisk walking, starting, stopping, avoiding obstacles, turns). During these exercises, participants are also exposed to a variety of cognitive tasks under dual-task conditions designed to tax specific executive functions and to challenge their focus of attention using acoustic and visual stimuli. Furthermore, exercises for strength, balance, and flexibility as well as endurance performance associated with walking are integrated (cf. Table 2).

There is a focus on everyday skills to promote ADL, cognition and psychosocial resources considering residents’ desires and preferences. For example, by using motivational equipment with different colors during the exercise sessions, a stimulating environment will be provided.

(2) *PROfit plus*

The main structure and contents of the *PROfit basic* intervention will be transferred into the *“PROfit plus” intervention*. In contrast to the basic intervention, the participants will perform the walking exercises throughout the facilities and attend predefined spots in the nursing home. There will be four areas where the activities (1. warm-up/mobilization; 2. coordination and balance; 3. muscle strengthening exercises; 4. cool-down) will be conducted. The walking part of the program will be used to reach these areas. The trainer will check if all participants walk a progressing distance comparable to the *PROfit basic* intervention.

(3) *PROfit orientation*

The *PROfit orientation* intervention integrates navigation and orientation tasks into the walking route including tasks for egocentric and allocentric orientation. The training integrates pointing to specific predefined landmarks (allocentric and egocentric reference frame), remembering the order of landmarks encountered along the route (route knowledge), and navigating to a specific room or area in the facility (planning and navigation). Other navigation and orientation tasks are integrated into the warm-up/mobilization and the coordination, balance, and executive function parts of the training, e.g. pointing or moving to cardinal directions (direction pointing) or assembling sections of the facility map like a puzzle (cognitive map).

**Fig. 2:**
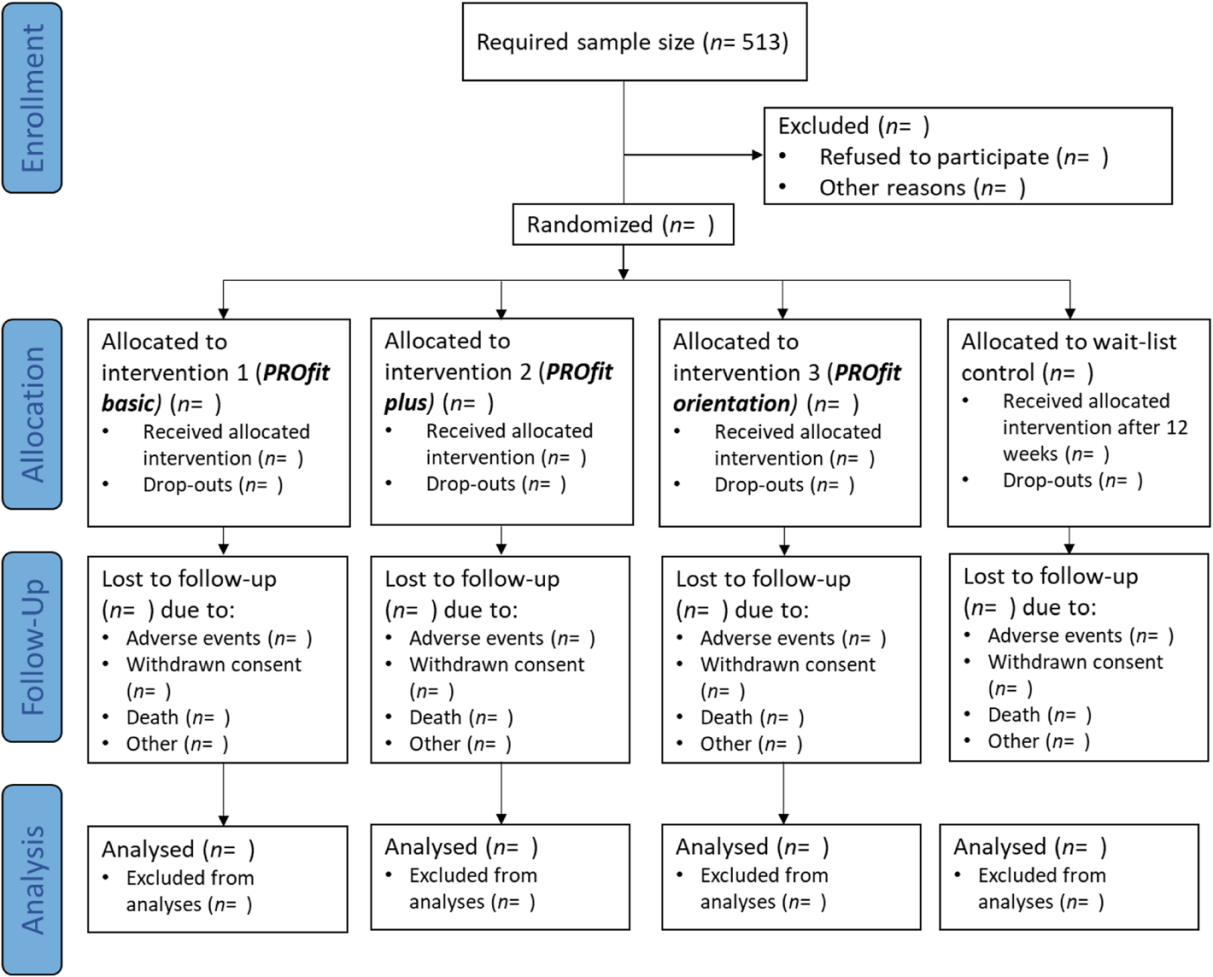
CONSORT flow diagram for PROfit

Overall, the exercise programs will be continuously adapted to the residents’ capacity and organized as a progressive challenge to expand participants’ resources. This will be done following the FITT principle (Frequency, Intensity, Time, Type) [94] and the recommendations of Herold and colleagues [39] to establish a cognitively beneficial program. These recommendations refer to a regularly performed sequence of structured and progressive physical exercises that are adapted to individuals’ performance limits [39]. Given the different pre-conditions of the participants, adaptions in different domains (e.g. physical or cognitive) are non-linear; trainers have to constantly adjust the program to participants’ abilities. Participants in the wait-list control group will be asked to continue their regular everyday activities for twelve weeks until they receive the intervention type which was randomly assigned to the nursing home (cf. Fig. 2).

### Data collection, management, and analysis

#### Statistical analysis

Descriptive data will be reported as group mean values and standard deviations or medians and interquartile ranges depending on the type of outcome and their distribution. Analysis of baseline differences will be performed using a multivariate analysis of variance (MANOVA). Parametrical and normally distributed measures will be analyzed in separate 4 (group: PROfit basic, PROfit plus, PROfit orientation, wait-list control group) × 3 (test: baseline assessment, post-allocation, close-out-testing) analyses of variance (ANOVA) with repeated measures. If baseline differences are detected, baseline assessment values as well as age and sex discrepancies will be included as covariates in the statistical model. In the case of statistically significant interaction effects, Bonferroni-adjusted post-hoc tests (t-tests, Wilcoxon tests) will be performed to detect statistically significant differences in the groups between baseline- and post-allocation-testing. Kruskal-Wallis and Friedman tests instead of ANOVA will be used for non-parametrical variables and for data for which normal distribution could not be assumed. The effect size will be determined using Cohen’s *f* [95] which is indicative of the effectiveness of treatment and helps to assess whether a statistically significant difference is of practical concern. Cohen’s *f* values are classified as small (0 ≤ *f* ≤ 0.24), medium (0.25 ≤ *f* ≤ 0.39), or large (*f* ≥ 0.40). Additionally, PS_dep_ scores (probability of superiority for dependent samples) will be computed as an estimate of effect size in non-parametrical post-hoc tests [96]. The significance level will be set at *p* < 0.025.

Intention-to-treat analysis will be conducted. For this purpose, data of all participants will be processed in the groups into which they were randomly assigned, regardless of whether they received or adhered to the assigned intervention. The majority of participants are expected to take part in a minimum of 80% of the training sessions. An additional per-protocol analysis will be performed if a sufficient number of participants are lost to outcome assessment or insufficient participation in training sessions. Multiple Imputation will be used to handle missing data assuming that data are missing by chance.

#### Sample size estimate / power calculations

The required sample size was calculated with G*Power (Version 3.1.9.2, Heinrich Heine University of Duesseldorf) [97]. The following input parameters were used to obtain small-sized test × group interaction effects: effect size (f = 0.15), type I error (α = 0.05), type II error (1-β = 0.95), number of groups (*n* = 4), number of measurements (*n* = 3), correlation between measurements (r = 0.60). Additionally, a dropout rate of 30% (i.e., 20% lost to follow-up; plus 10% deceased) was considered. Our analysis resulted in a total sample size of 513 participants (i.e., 171 per center with 42–43 participants allocated to each group).

#### Monitoring

A data monitoring committee responsible for data monitoring, interim analyses, and auditing will be established. Project staff will intervene if negative reactions are observed during assessments and training interventions. Grant holders are part of a PROfit advisory board and responsible for data audits every 6 months.

## Discussion

The overall goal of this multicentric RCT is to determine the effects of three different cognitive-motor training interventions on PA, LS, and spatial orientation in a sample of nursing home residents. We expect slower declines or even improvements in PA, LS mobility, and spatial orientation in participants of all three groups compared to wait-list controls. Regarding the secondary outcomes, we expect that physical functioning, cognitive functioning, and psychosocial well-being will be improved in all residents participating in one of the three intervention arms, when compared to the wait-list control group.

The interventions of the PROfit program combine components of exercise programs that have been proven to yield health benefits for nursing home residents [51, 93, 98, 99]. Especially the physical outcomes, e.g., walking capacity, muscle strength, and balance capacity have been shown to be improved by multicomponent interventions as conducted within the PROfit approach [91, 93, 100].

However, regarding PA and LS, effectiveness has not yet been investigated. Therefore, we extended previous intervention approaches with changes of location during training and specific spatial orientation training to follow the principles of training and task specificity to increase the training effects. By adding different locations where the training is administered, we aim to increase physical, cognitive, orientation and psychosocial parameters substantially more with *PROfit plus* than with *PROfit basic.* Moreover, we expect the *PROfit orientation* intervention to be most effective due to its additional navigation and spatial orientation training components. We suppose that the effects of *PROfit orientation* on the primary outcomes will be reached via two mechanisms: (1) increased physical and cognitive performance will enhance cognitive-motor capacities and (2), according to Vance and colleagues the spatial training will help to build up cognitive strategies to compensate for age-related losses and limitations [64]. This, in turn, could translate into enhanced PA and LS due to better abilities to find the way, better knowledge of the nursing home building, alleys, floors, and room plans. One cognitive strategy would be the use of newly learned landmarks for an egocentric-based route strategy. Here, landmarks can be used as indicators where to turn in which direction to reach a specific goal location. Alternatively, landmarks can be integrated into an allocentric representation of the environment leading to improved knowledge of relative directions between different locations or even a survey-like representation of the environment. Introducing existing objects in and near the facility as landmarks for orientation reflects a compensatory approach [65] which in turn allows training of different spatial strategies based on this new information, eventually leading to improved spatial orienting abilities.

Overall, potential ways to encourage nursing home residents to participate actively in social life within the care setting are provided by facilitating a program that is appropriate and adapted to residents’ capacities, needs, and desires. Moreover, introducing a specific spatial cognitive component to the program and investigating the impact of nursing home facilities’ spatial structure and landmark availability, this program will allow to develop guidelines for interventions that specifically increase spatial orientation abilities in care home facilities and their contribution to general LS mobility and well-being. Results from this trial will particularly contribute to the evidence on motor-cognitive approaches in the maintenance of mental and physical functioning.

To this end, the findings may provide suggestions and support to handle present and future challenges, occurring at health promotion initiatives in the setting of long-term care, a sector that is highly relevant in times of aging populations in Western societies. With the prevention act of 2015, German health insurances have to provide preventive services in nursing homes. The multicentric RCT will show that universal prevention through motor exercise and spatial orientation training is possible and useful to improve health status and personal resources of nursing home residents.

## Data Availability

All participant information and data will be stored securely and identified by a coded ID number only to maintain participants’ confidentiality. It is planned to transfer the data to an open access repository.

## Abbreviations

ADL: Activities of daily living
BMI: Body Mass Index
COF: Concerns of falling
DIA-S: Depression in Age Scale
DRKS: German Clinical Trials Register
FITT: Frequency, intensity, time and type of exercise
GCP: Good Clinical Practice
IADL: Instrumental activities of daily living
IAGG: International Association of Gerontology and Geriatrics
LS: Life-space
Mini-BESTest: Mini Balance Evaluation Systems Test
MoCA: Montreal Cognitive Assessment
iTUG: Smartphone-based instrumented Timed Up and Go Test
NHLSD: Nursing Home Life-Space Diameter
PA: Physical Activity
PROfit: Prevention Orientation Training
RCT: Randomized controlled trial
SD: Standard deviation
Short FES-I: Short Falls Efficacy Scale international
SPIRIT: Standard Protocol Items: Recommendations for Interventional Trials
SPPB: Short Physical Performance Battery
SWLS: Satisfaction with Life Scale
